# Quantifying the role of social distancing, personal protection and case detection in mitigating COVID-19 outbreak in Ontario, Canada

**DOI:** 10.1186/s13362-020-00083-3

**Published:** 2020-05-26

**Authors:** Jianhong Wu, Biao Tang, Nicola Luigi Bragazzi, Kyeongah Nah, Zachary McCarthy

**Affiliations:** 1 Laboratory for Industrial and Applied Mathematics, Department of Mathematics and Statistics, York University, Toronto, Canada; 2 Fields-CQAM Laboratory of Mathematics for Public Health, York University, Toronto, Canada

**Keywords:** COVID-19, Personal protection, Mathematical model, Control reproduction number, Effective reproduction number, Parameter estimation

## Abstract

Public health interventions have been implemented to mitigate the spread of coronavirus disease 2019 (COVID-19) in Ontario, Canada; however, the quantification of their effectiveness remains to be done and is important to determine if some of the social distancing measures can be relaxed without resulting in a second wave. We aim to equip local public health decision- and policy-makers with mathematical model-based quantification of implemented public health measures and estimation of the trend of COVID-19 in Ontario to inform future actions in terms of outbreak control and de-escalation of social distancing. Our estimates confirm that (1) social distancing measures have helped mitigate transmission by reducing daily infection contact rate, but the disease transmission probability per contact remains as high as 0.145 and case detection rate was so low that the effective reproduction number remained higher than the threshold for disease control until the closure of non-essential business in the Province; (2) improvement in case detection rate and closure of non-essential business had resulted in further reduction of the effective control number to under the threshold. We predict the number of confirmed cases according to different control efficacies including a combination of reducing further contact rates and transmission probability per contact. We show that improved case detection rate plays a decisive role to reduce the effective reproduction number, and there is still much room in terms of improving personal protection measures to compensate for the strict social distancing measures.

## Introduction

Coronaviruses are enveloped, single-stranded, positive-sense RNA viruses that usually cause mild respiratory communicable disorders but can, sometimes, result in a severe and even lethal infection [1]. Coronaviruses are considered re-emerging pathogens, due to globalization, increasing urbanization, frequency of contacts and mixing of various animals in high-density areas [2, 3]. Due to their highly dynamic mutation and recombination rates and their ability to cross species, they can adapt to new hosts [4, 5], and, as such, in the last decades they have caused different large-scale outbreaks and zoonotic spillovers, including the 2003 “Severe Acute Respiratory Syndrome” (SARS) outbreak, and the “Middle East Respiratory Syndrome” (MERS) outbreaks occurred in 2012 in the Kingdom of Saudi Arabia and in 2015 in South Korea [5].

The “Severe Acute Respiratory Syndrome-related Coronavirus type 2” (SARS-CoV-2), initially termed as 2019-nCoV, is an emerging coronavirus, which has caused the latest coronavirus outbreak, which from the first reported epicenter, Wuhan (province of Hubei, People’s Republic of China), has spread out globally, becoming a pandemic [1]. SARS-CoV-2 is highly contagious and quickly spreads among individuals, causing an infection in which the severity varies from asymptomatic cases to a life-threatening disorder, known as “coronavirus disease 19” (COVID-19) [1]. China adopted a package of strict public health measures [6–8], including quarantine and lock-down of entire regions, these interventions may be considered unfeasible/unsustainable in other countries, including the western societies, which have preferred a mitigation- rather than a suppression-based strategy. Canada belongs to the countries which have chosen to mitigate the burden imposed by the viral outbreak. In Canada, the first confirmed COVID-19 case was identified on January 25th 2020. As of April 1st 2020, Canada has reported a total of 11,283 cases, with 173 deaths, including 2392 confirmed cases in Ontario. The Canadian government and the province of Ontario has gradually implemented and increasingly enhanced a package of public health control measures, including travel restrictions, closure of schools, universities and several business practices. Approximately one third of reported cases are travel-related, with most of them being related to local transmission.

While controls have been implemented, their efficacy and the future trend of COVID-19 in Ontario is uncertain. We aim to equip local public health decision- and policy-makers with mathematical model-based estimation of mitigation measure efficacy and projection of the trend of the COVID-19 epidemic in Ontario, in order to inform dynamic optimization of ad hoc measures in a fast developing epidemic. In particular, we develop a transmission model taking full consideration of the mitigation strategies implemented in Ontario: physical distancing, contact tracing and diagnosis. We parameterize this transmission model by fitting to the reported incidence data. Using this parameterized model, we assess the transmission risk and evaluate the effectiveness of interventions.

## Material and methods

### Model

**Figure 1:**
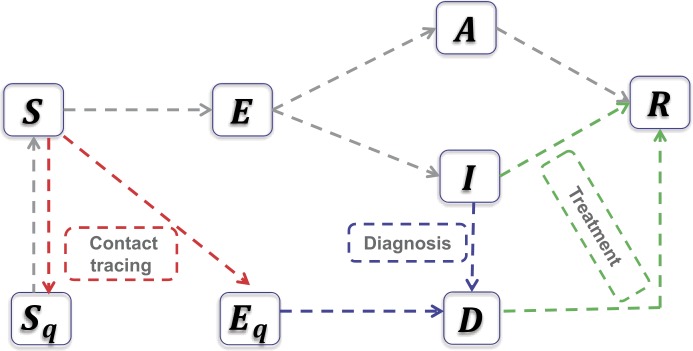
An illustration of the model describing the transmission of novel coronavirus (COVID-19) infection under control measures-contact tracing and isolation (red line), diagnosis (blue line) and treatments (green line). The fundamental model framework can refer to the studies [6, 7]

We use the modeling framework of COVID-19 transmission developed in previous studies [6–8] to describe the COVID-19 transmission dynamics in Ontario, Canada. The population is divided into susceptible (*S*), exposed (*E*), asymptomatic infectious (*A*), infectious with symptoms (*I*), and recovered (*R*) compartments according to the epidemiological status of individuals, and further into diagnosed (*D*), quarantined susceptible ($S_{q}$), and isolated exposed ($E_{q}$) compartments based on control interventions. We also account for contact tracing, where a proportion, q, of individuals exposed to the virus are quarantined. The quarantined individuals can either move to the compartment $E_{q}$ or $S_{q}$, depending on whether they are effectively infected or not, while the other proportion, $1-q$, consists of individuals exposed to the virus who are missed from contact tracing and, therefore, move to the exposed compartment E once effectively infected, or stay in the compartment *S* otherwise [6]. The transmission diagram is shown in Fig. 1 and the transmission dynamics model is given by [7]

1 $$ \begin{gathered} S'=-\bigl(\beta c+cq(1-\beta )\bigr)S(I+ \theta A)/N+\lambda S_{q}, \\ E'=\beta c(1-q)S(I+\theta A)/N-\sigma E, \\ I'=\sigma \rho E-(\delta _{I}+\alpha +\gamma _{I})I, \\ A'=\sigma (1-\rho )E-\gamma _{A} A, \\ S_{q}'=(1-\beta )cqS(I+\theta A)/N-\lambda S_{q}, \\ E_{q}'=\beta cqS(I+\theta A)/N-\delta _{q} E_{q}, \\ D'=\delta _{I} I+\delta _{q} E_{q}-( \alpha +\gamma _{H})D, \\ R'=\gamma _{I} I+\gamma _{A} A+\gamma _{H} D. \end{gathered} $$

where $N=S+E+I+A+S_{q}+E_{q}+D+R$ is the total population, and $N'=-\alpha (I+D)$. The definitions of all the parameters are listed in Table 1. Using the next generation matrix method [9], we obtain the control reproduction number of model (1) as the following:

$$ R_{c}=\bigl(\beta \rho c(1-q)\bigr)/(\delta _{I}+\alpha + \gamma _{I} )+\bigl(\beta c \theta (1-\rho ) (1-q)\bigr)/\gamma _{A}. $$

Here, the control reproduction number means the basic reproduction number with control interventions as the Ontario government adapted a series of control measures since March 14.

**Table 1:** Parameter estimates for COVID-19 in Ontario, Canada

Parameter		Definitions	Feb 26 to Mar 21	Feb 26 to Mar 25	Feb 26 to Mar 29	Feb 26 to Apr 13	Source
*c*		Contact rate	11.7905	11.5539	9.1586	–	Estimated
*c*(*t*)	$c_{0}$	Constant contact rate before $T_{s}$	–	–	–	10.0005	Estimated
	$r_{1}$	Exponential decreasing rate of contact rate	–	–	–	0.0632	Estimated
	$c_{b}$	Minimum contact rate after $T_{s}$	–	–	–	3.4999	Estimated
*β*		Probability of transmission per contact	0.1450	0.1450	0.1452	0.1438	Estimated
*q*		Quarantined rate of exposed individuals	0.0810	0.1479	0.1754	0.1003	Estimated
*σ*		Transition rate of exposed individuals to the infected class	1/5	1/5	1/5	1/5	[10]
*λ*		Rate at which the quarantined uninfected contacts were released into the wider community	1/14	1/14	1/14	1/14	[7]
*ρ*		Probability of having symptoms among infected individuals	0.6	0.6	0.7847	0.6201	Estimated
$\delta _{I}$		Transition rate of symptomatic infected individuals to the quarantined infected class	0.1	0.1	0.1	–	Estimated
$\delta _{I}(t)$	$\delta _{I0}$	Constant transition rate of symptomatic infected individuals to the quarantined infected class before $T_{s}$	–	–	–	1/9.2	Data
	$r_{2}$	Exponential increasing rate of the detection rate	–	–	–	0.7174	Estimated
	$\delta _{If}$	Fastest transition rate of symptomatic infected individuals to the quarantined infected class after $T_{s}$	–	–	–	0.5642	Estimated
$\delta _{q}$		Transition rate of quarantined exposed individuals to the quarantined infected class	0.1	0.1	0.1	0.1	Estimated
$\gamma _{I}$		Recovery rate of symptomatic infected individuals	0.2	0.2	0.1999	0.1830	Estimated
$\gamma _{A}$		Recovery rate of asymptomatic infected individuals	0.139	0.139	0.139	0.139	[7]
$\gamma _{H}$		Recovery rate of quarantined diagnosed individuals	0.2	0.2	0.2	0.2	[6]
*α*		Disease-induced death rate	0.008	0.008	0.008	0.008	[6]
*θ*		Modification factor of asymptomatic infectiousness	0.0429	0.0465	0.0308	0.0494	Estimated
$R_{c}$		Control reproduction number	3.2546	2.9720	2.8464	–	Estimated

Initial values	Definitions	Feb 26 to Mar 21	Feb 26 to Mar 25	Feb 26 to Mar 29	Feb 26 to Apr 13	Source
*S*(0)	Initial susceptible population	1.999 × 10^6^	1.999 × 10^6^	1.999 × 10^6^	1.993 × 10^6^	Estimated
*E*(0)	Initial exposed population	10	12	10	10	Estimated
*I*(0)	Initial symptomatic infected population	7.6	7	8.5	10	Estimated
*A*(0)	Initial asymptomatic infected population	15	24	23	24	Estimated
$S_{q}(0)$	Initial quarantined susceptible population	0	0	0	0	Data
$E_{q}(0)$	Initial quarantined exposed population	0	0	0	0	Data
*D*(0)	Initial quarantined diagnosed population	5	5	5	5	Data
*R*(0)	Initial recovered population	0	0	0	0	Data

### Data and parameter estimation process

**Figure 2:**
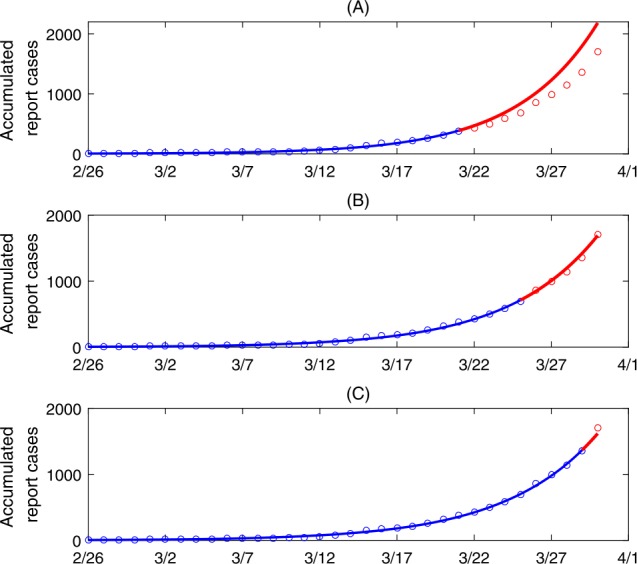
Best model fitting results and model predictions. Here, the blue curves are the best fitting curves while the red curves are the predicted curves from the best fitting model. The circles denote the real data

We obtained the data of cumulative reported COVID-19 infected cases in Ontario, Canada, from the Government of Canada [11], as shown in Fig. 2. The data were released and analyzed anonymously.

When carrying out the parameter estimations, we fixed several of the model parameters from the literature or based on the available information in order to reduce the complexity of the parameter space needing estimation. In detail, the incubation period is fixed as 5 days [10], i.e. $\sigma =1/5$, the rate at which the quarantined uninfected contacts were released into the wider community is fixed as $\lambda =1/14$ [7], while the recovery rate of the asymptomatic infections is fixed as $\gamma _{A}=0.139$ [7]. As we did not use the data of death cases and the recovered population, we fixed the recovery rate of the diagnosed population as $\gamma _{D}=0.2$ and the disease-induced death rate as $\alpha =0.008$ following the estimations in the study [6]. Based on the data information, the initial quarantined suspected population, quarantined exposed population, and the recovered population were all set as 0, while the initial diagnosed population is fixed as 5. In this study, we used the least square method with *a priori* distribution for each parameter to fit the model to the confirmed cases data using Matlab. The objective function is defined as the residual sum of squares between the real data of the time series of cumulative reported cases and the predicted number by solve system (1), which is solved by the “ODE45” function. And the “fmincon” function in Matlab is used to search the optimal solutions. The seminal work on social contact modelling (under normal societal conditions) from Mossong et al. found that 7290 study participants had an average of 13.4 contacts per day per person [12]. The estimated contact rate in our study, we expected to be lower than this count, as the model definition for contact rate is contacts per day among the participating group of individuals in Ontario. Therefore, we account for *a priori* contact information and set the bounds of contact rate as $(10,14)$ in the first phase with insignificant public health control interventions. As a series of control measures were taken, we do believe that there was a decreasing of the contact rate, and we set the bounds on the contact rate interval to $(7,13)$.

## Results

Retroactive analysis: Using the least squares method, we fitted the model to the data of cumulative reported cases in Ontario, Canada, during the periods from February 26th to March 21st (Fig. 2(A)), February 26th and March 25th (Fig. 2(B)), and February 26th to March 29th (Fig. 2(C)), respectively. The best fit parameters and initial conditions are listed in Table 1.

We estimated the control reproduction number of the COVID-19 epidemic in Ontario to be 3.25 between February 26 and March 21, 2.97 between February 26 and March 25, and 2.84 between February 26 and March 29 (Table 1). Other estimated parameter values measuring the effectiveness of contact tracing, quarantine, testing average daily contact rates, and transmission efficacy per contact will be described in next section to identify important gaps in effective mitigation.

**Figure 3:**
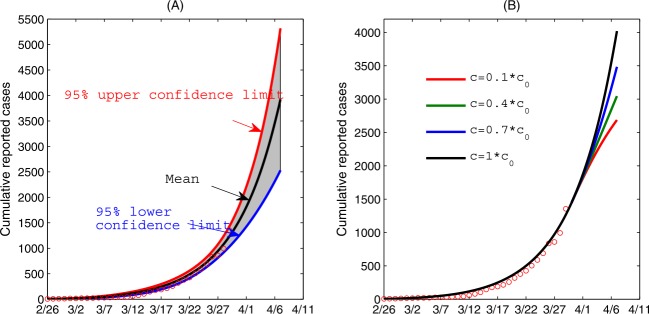
(**A**) The impact of the randomness of the data of cumulative confirmed cases on the epidemics in Ontario. (**B**) The impact of contact rate on the cumulative confirmed cases, where we assumed that the contact rate decreases since March 30th. Here, $c_{0}$ is the estimated value based on the fitting results in (**A**)

We also made a short-term prediction on the cumulative reported cases until April 7, based on the parameter identification using data until March 29. We used this short-term projection to demonstrate the impact of randomness of data on the COVID-19 epidemic in Ontario, shown in Fig. 3(A). Here, we assumed that the number of confirmed cases follows a Poisson distribution with mean given by the reported number. We generated 500 cumulative incidence data sets and re-estimated model parameters for each data set. We utilized this procedure to provide the mean and 95% confidence interval in our projection until April 7th. We found that the number of the reported cases keep a fast-increasing trend in Ontario, and the predicted number of cumulative confirmed cases is 6132 (95% CI 4250–8000) as of April 7th without an indication of approaching epidemic peak, suggesting enhanced mitigation measures must be taken. We also conducted a sensitivity analysis showing how further decreasing the contact rate since March 30th would affect the cumulative reported cases, shown in Fig. 3(B). In particular, we observed that the cumulative reported cases decrease significantly as the contact rate is further decrease: the additional number of the cumulative reported cases between March 30th and April 7th can decrease by $\sim 50\%$ when the contact rate decreases by 90%.

**Figure 4:**
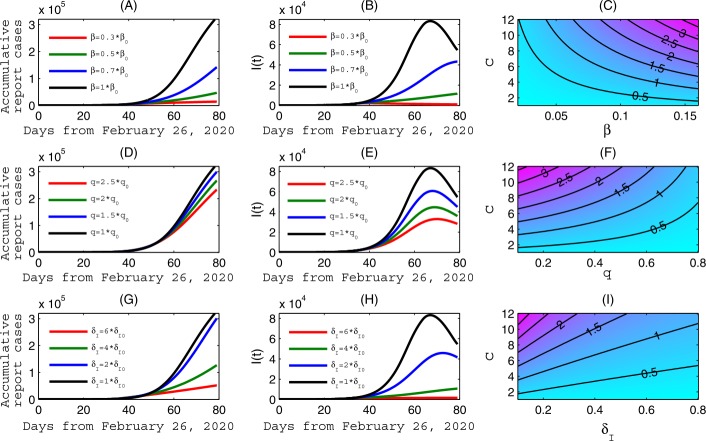
Sensitivity analysis. The impact of the transmission probability per contact *β* in (**A**–**B**), the quarantine rate *q* in (**D**–**E**), and the diagnose rate $\delta _{I}$ in (**G**–**H**), respectively, on the COVID-19 epidemics of Ontario. (**C**), (**F**) and (**I**), Contour plots of the control reproduction number with respect to the contact rate *c* and the transmission rate *β*, or quarantine rate *q*, or rate diagnose rate $\delta _{I}$, respectively. Here, the baseline values of the parameters are fixed as the same as those in Fig. 3, $\beta _{0}$, $q_{0}$ and $\delta _{I0}$ denote the estimated values

However, the reduction of transmission contact rate will have a limit. On the other hand, our estimation of the transmission probability per contact, *β*, has been virtually unchanged since February 26th (0.145) indicating lack of improvement of personal protection. We show, in Fig. 4(A–B), that the cumulative reported cases and the infected population at the peak time decrease significantly as *β* is reduced. Particularly, the epidemics in Ontario could have peaked around April 2 should the transmission probability have been decreased by 70%. Similarly, we can reduce the cumulative reported cases and peak value of infected population by increasing the quarantine rate *q* and, importantly, the diagnose rate $\delta _{I}$, as shown in Fig. 4(D–E) and Fig. 4(G–H), respectively. The contour plots of the control reproduction number in Fig. 4(C), (F) and (I) show that the control reproduction number can be reduced to the threshold 1, in a very short period of time, when a combination of reduction of transmission contact rate and transmission probability is implemented, or a combination of increasing the quarantine rate and increasing the diagnosis rate. This analysis, proactively done on March 29, is relevant for planning the de-escalation of social distancing measures as it shows what kind of synergistic combination of social distancing measures will be needed to counteract future outbreaks potentially caused by relaxation of social distancing.

Real-time analytic and projection: An important insight gained from the above retroactive analysis based on data in different phases between February 26 to March 29 is that intervention efficacy has been evolving as the public health measures have been escalated. In particular, we noted the gradual decreasing of contact rate, and hence the control reproduction number. Since March 24, the province of Ontario has taken some additional public health measures to include the closure of any non-essential business and the improvement of testing to increase the detection rate. As shown in the previous studies [6, 8], a more appropriate way to reflect these two major measures is to adopt a non-autonomous transmission dynamics model with time-dependent contact rate and case detection rate as follows:

2 $$ c(t)= \textstyle\begin{cases} c_{0},&t< T_{s}, \\ (c_{0}-c_{b} )e^{-r_{1} (t-T_{s})}+c_{b},& t\geq T_{s} \end{cases} $$

and

3 $$ \delta _{I}(t)= \textstyle\begin{cases} \delta _{I0},&t< T_{s}, \\ (\delta _{I0}-\delta _{If})e^{-r_{2} (t-T_{s})}+\delta _{If},& t\geq T_{s}, \end{cases} $$

where $c_{0}$ is the constant contact rate and $\delta _{I0}$ denotes the constant detection rate before the time $T_{s}$. Here, $T_{s}=27$ (corresponding to March 24, when non-essential business was closed and case detection rate was increased). We assume the contact rate $c(t)$ began to decrease at an exponential rate $r_{1}$ while the detection rate $\delta _{I} (t)$ began to increase at an exponential rate of $r_{2}$. $c_{b}$ (to be estimated) is the minimum contact rate that such a non-essential business closure can achieve) and $\delta _{If}$ is the maximum detection rate with the current testing capacity. Based on the formula of the control reproduction number, we can further define the effective (daily) reproduction number replacing the constant contact rate *c* and the diagnose rate $\delta _{I}$ with the functions defined in Eqs. (2) and (3). Therefore, the effective reproduction number is time-dependent. It should be mentioned that there should be the $S(0)/N(0)$ term in the formula of the control reproduction number with $S(0)/N(0)=1$. Similarly, we assume that $S(t)/N(t)\approx 1$ as the susceptible population size is very large. Then, the effective reproduction number can be defined as follows:

$$ R_{t}=\bigl(\beta \rho c(t) (1-q)\bigr)/\bigl(\delta _{I} (t)+\alpha +\gamma _{I} \bigr)+\bigl( \beta c(t)\theta (1-\rho ) (1-q) \bigr)/\gamma _{A} . $$

Note that with constant contact rate *c* and the diagnose rate $\delta _{I}$, the effective reproduction number coincides with the control reproduction number, $R_{c}$. The above-defined effective reproduction number is an estimate for the average number of secondary cases per primary case introduced at time *t*, assuming that the contact rate and the rate of diagnosis would remain constant at the level of $c(t)$ and $\delta _{I}(t)$ during one’s infectious period, also assuming that the depletion of susceptible persons during the epidemic is negligible.

**Figure 5:**
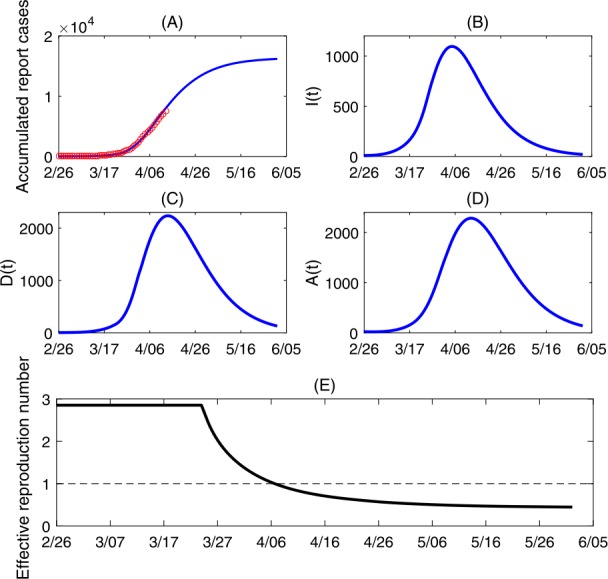
(**A**) Best model fitting result; (**B**–**D**) Solutions of model (1) by fixing the parameters as the estimated values; (**E**) Estimated effective reproduction number of Ontario

We then re-fitted the autonomous model to the data between February 26 to April 13, and re-estimated the parameter values, listed in Table 1 as well. The best fitting result is shown in Fig. 5(A). We also plotted the solutions of $I(t)$, $D(t)$ and $A(t)$ in Fig. 5(B–D) by fixing the parameters as estimated. It follows from Fig. 5(C) that the time series of reported cases has passed the peak around April 12. Further, we showed in Fig. 5(E) that the effective reproduction number of Ontario decreased to below the threshold 1 around April 7. This is in line with our sensitivity analysis and predictions in last subsection that, based on the estimation using the earlier epidemic data, conclude that reducing contacts and improving the case detection rate can be an effective way in controlling the COVID-19 epidemics.

We implicitly utilized a constrained least square method to select the parameter values identified through the least square method which collectively fall in the ranges estimated in other published studies that are described below. The recovery rate of those symptomatic and infectious, in our model, can also be interpreted as the baseline infectious period of those presenting symptoms. Our estimate rate of $\gamma _{I}=0.183-0.2\mbox{ 1/day}$ correspond to an average infectious period of 5 to 5.5 days which is consistent with those adopted by prior studies, such as [13]. We expected our estimated contact rates to be lower than those from social contact surveys as a result of factors including the public’s heightened awareness from declaration of the pandemic in addition to public health interventions implemented by the province [14]. The final contact rate $c_{b}$ we estimated of 3 is slightly above the average rates from contact surveys collected in Shanghai, China and Wuhan, China during the (stricter) lockdown [15]. As expected, the estimated contact rate was lower in China than in Ontario, since the lockdown was stricter. The diagnosis rate $\delta _{I}$ also has the interpretation of the average time from symptom onset to case diagnosis among those symptomatic individuals. Hence, our estimated rate of $\delta _{I}= 0.1\text{ day} ^{-1}$ are indicative of an average 10 days from symptom onset to diagnosis. This rate estimated is in line with our direct calculation of an average of 9.2 days from onset to case report using the individual line-list of all documented and confirmed COVID-19 cases in Ontario. Furthermore, the diagnosis rate among those quarantined and exposed to the virus can be interpreted as the average time from exposure (transmission event) to diagnosis. Hence, our estimates of $\delta _{q}= 0.1\text{ day} ^{-1}$ are indicative of 10 days from exposure to diagnosed among those quarantined. In light of the disease’s estimated average incubation time of 5 days, we then estimated an average of 5 days for individuals to seek health care and have their test confirmed. This finding is logical, as we estimated quarantined individuals were confirmed (on average) substantially faster than those not quarantined. In other words, we captured the heightened awareness of those individuals quarantined in our estimated value of $\delta _{q}$. The estimated final diagnosis rate $\delta _{If}$ among symptomatic individuals qualitatively captured the effects of enhanced testing throughput, which was implemented by the province in late March, on case detection time.

## Discussion

Ontario has been escalating a series of public health measures aimed at containing the COVID-19 outbreak. As of March 14th, Ontario closed all public schools until April 5th. On March 16th, the Ministry of Health requested closure of all recreational programs and libraries, all private schools, all daycares, all churches and other faith settings, all bars and restaurants, with the exception of those that can shift to a takeout/delivery mechanism. On March 17th, Ontario declared state of emergency and, after an initial transition, as of March 24th at midnight, closure of all non-essential workplaces becomes mandatory and effective.

In the present study, using the COVID-19 incidence report data from February 26th to April 13, we estimated key epidemiological and intervention parameters of a disease transmission model in Ontario. We estimated the transmission risk and studied the impact of public health interventions implemented by Ontario, we also estimated the probability of individuals presenting symptoms, which is consistent with previously established estimates [16]. While the control reproduction number had been decreasing with time, our simulations based on data as of March 29 indicated that further compliance with measures and additional interventions were needed to reduce below the control threshold of 1. We pointed out that a combination of additional reduction of contacts and increasing the detection rate would help to reduce the effective reproduction number to under the unity for disease control, and our estimation and analysis based on time series of cumulative reported cases since March 24 confirmed this strategy worked: since the closure of non-essential business and increasing the detection rate since March 24, we noted rapid decline of the effective reproduction number and the fast approaching of the epidemic peaks.

In particular, to identify the evolution of contact rates and other parameters potentially affected by the variation of intervention intensity, we have estimated the relevant parameters using cumulative reported cases by March 21st, March 25th, and by March 29th, respectively. As a result, the control reproduction numbers estimated using the different time intervals decreased from 3.25 to 2.97 and 2.84, indicating a gradually increasing effectiveness of the interventions adopted. Further, we illustrated how the control interventions adopted by the government and the public have begun to play an important role in mitigating the epidemic in Ontario since March 21st (Fig. 2). Using the estimated parameters and based on the model, we predicted the number of cumulative confirmed cases as of April 7th to be 6132 (95% CI 4250–8000) (Fig. 3(A)), and we noted that this could have been further reduced by $\sim 50\%$ by decreasing the contact rates from March 30th to April 7th by 90% (Fig. 3(B)). Hence the level of compliance with social distancing advisories by the public and the increasing capacity of case detection can influence the future trend of the epidemic.

Comparing the estimates from the three different time intervals before the closure of non-essential business, we conclude that the intervention efforts and control measures, such as lockdown and social distancing advisories, was effective in reducing the person-to-person contact rate (Table 1). We also estimated that proportionally more cases are being quarantined which had contributed to the decrease of the control reproduction number (Table 1). However, the estimated transmission efficacy was not changed significantly since the adoption of controls (Table 1) until March 24, indicating that the intensity of personal protections against effective contacts was not improved. Although the control reproduction number by March 24 had decreased, it was still close to 3 and would be below 1 only if the effective contact rate (the contact rate times the transmission probability per contact) is reduced by 2/3. Therefore, there should be persistent control efforts in reducing contact rate and the duration of infection of infectious individuals, which is possible with a fast case detection rate and effective isolation measures. We confirmed that this was indeed achieved two weeks after the closure of non-essential business and the improved testing.

We also considered the scenario that some of the efforts might have reached their maximum level by social distancing and self-isolation recommendations before the closure of non-essential business on March 24, so decreasing transmission efficacy by boosting personal protection could be a mitigation option. Using simulations, we illustrated the impact of reducing transmission efficacy on future cumulative number of confirmed cases, epidemic peak time, and peak number of infections as shown in Fig. 4(A) and Fig. 4(B). We also simulated the role of increasing testing which leads to increase in the case confirmation rate and suggested the possibility of a combination of rapid testing and effective quarantine (Fig. 4(D, E, F)) in reducing the case number and reducing the control reproduction number. Overall, the contact rate, transmission probability, detection/diagnose rate, and quarantine rate are key factors that influence the control reproduction number (Fig. 4(C), Fig. 4(F) and Fig. 4(I)).

While epidemic control in Ontario was achieved with reduction of contacts and increase of detection rate during the period from March 24 to April 13 as shown in Fig. 5, our simulations show that there is still much room for improvement in personal protection. This is an encouraging news for Ontarians to plan for their social distancing de-escalation strategy, as increasing personal protection may compensate a certain degree of social distancing relaxation. Social distancing advisories, closures, among the package of implemented control measures have assisted in reducing disease transmission; however, personal protection may admit further reduction in forward transmission. Personal protection in the form of improved hygiene such as handwashing, reduction of face-touching, and the appropriate utilization of personal protective equipment (PPE) offers the convenience of being actionable by all individuals in the public and may mitigate future COVID-19 transmission in Ontario. Mask wearing and instant hand wiping has been proposed and supported to mitigate transmission of COVID-19, and recent findings indicate that surgical face masks could prevent transmission of human coronaviruses from symptomatic individuals [14, 17]. In this light, Ontarians should intensify its effort in acquiring protective supplies such as masks and protective clothing, and develop an optimal distribution strategy if supplies are in insufficient supply.

Respiratory infectious diseases, such as COVID-19, are spread through a susceptible individual’s contact with infectious agents. These contacts facilitate disease transmission and can be made indirectly through environmental routes or through direct person-to-person interaction. PPE, such as surgical and N95 masks, are utilized with the aim of reducing the likelihood of disease transmission either from an asymptomatic infection to susceptible contacts, or upon an individual’s exposure to an infectious agent such as a cough or sneeze carrying a pathogenic load. However, as in the case of surgical and N95 masks, PPE may not be tested or efficacious for conditions which they may see in practice, including a range of plausible human respiratory emissions containing pathogenic loads [18, 19]. Further, no studies have directly evaluated the biophysics of droplets and gas formation for patients infected with the SARS-CoV-2 [18]. Lastly, in some regions, the availability of surgical masks may be limited in light of the high demand and recent production decreases [20]. All these points emphasize the urgent need for a mechanistic and comprehensive understanding of respiratory disease transmission, in order to effectively design PPE and enhance COVID-19 mitigation strategies such as social distancing. These points highlight several of the current challenges and difficulties of PPE adequately reducing disease transmission, warranting further research.

In conclusion, through the parametrization and simulation of disease transmission models with intervention mechanisms informed with multiple data sources including regional demographics, and regional intervention features, our work utilizes a foundational framework for intervention evaluation and intervention scenario analysis. This study highlights the opportunity for evaluation of control measures and trend in disease transmission to inform the future decision-making for preparedness, real-time management as well as risk assessment of COVID-19.
